# The effect of temperature and invasive alien predator on genetic and phenotypic variation in the damselfly *Ischnura elegans*: cross-latitude comparison

**DOI:** 10.1186/s12983-023-00494-z

**Published:** 2023-04-10

**Authors:** Guillaume Wos, Gemma Palomar, Marzena Marszałek, Wiesław Babik, Szymon Sniegula

**Affiliations:** 1 Institute of Nature Conservation Polish Academy of Sciences, al. Adama Mickiewicza 33, 31-120 Kraków, Poland; 2 Department of Genetics, Physiology, and Microbiology, Complutense University of Madrid, C/Jose Antonio Novais 12, 28040 Madrid, Spain; 3 Institute of Environmental Sciences, Jagiellonian University, Gronostajowa 7, 30-387 Kraków, Poland

**Keywords:** Gene expression analysis, Global changes, Human disturbance, Invasive alien species, *Ischnura elegans*, Latitude, Life history, Multivariate analysis

## Abstract

**Background:**

Understanding and predicting how organisms respond to human-caused environmental changes has become a major concern in conservation biology. Here, we linked gene expression and phenotypic data to identify candidate genes underlying existing phenotypic trait differentiation under individual and combined environmental variables. For this purpose, we used the damselfly *Ischnura elegans*. Egg clutches from replicated high- (southern Sweden) and central-latitude (southern Poland) populations facing different degrees of seasonal time constraints were collected. Damselfly larvae were exposed to experimental treatments: current and mild warming temperatures crossed with the presence or absence of an invasive alien predator cue released by the spiny-cheek crayfish, *Faxonius limosus*, which is only present in Poland to date. We measured the following traits: larval development time, body size, mass and growth rate, and used the larvae for gene expression analysis by RNA-seq. Data were analysed using a multivariate approach.

**Results:**

We showed latitudinal differences in coping with mild warming and predator cues. When exposed to an increased temperature and a predator cue, central-latitude individuals had the shortest development and the fastest growth compared to high-latitude individuals. There was a general effect of predator cues regarding mass and growth rate reduction independent of latitude. Transcriptome analysis revealed that metabolic pathways related to larval anatomy and development tended to be upregulated in response to mild warming but only in fast-growing central-latitude individuals. Metabolic pathways linked to oxidative stress tended to be downregulated in response to a predator cue, especially in central-latitude individuals.

**Conclusion:**

Different phenotypic and transcriptomic responses to environmental factors might be attributed to the variability in *I. elegans* life history strategies between the two latitudes caused by seasonal time constraints and to its coexistence with the invasive alien predator in nature. By providing insights into how organisms may respond to future anthropogenic changes, our results may be of particular interest in conservation biology.

**Supplementary Information:**

The online version contains supplementary material available at 10.1186/s12983-023-00494-z.

## Background

Increasing anthropogenic pressures pose a serious threat to natural ecosystems. Understanding and predicting how organisms respond to human-caused environmental changes has become a major concern in conservation biology. Global warming, habitat degradation, pollutants or biological invasions are often considered the main threats causing a rapid decline and/or shift in animal communities [1–3]. Much effort has been made to assess the effects of anthropogenic disturbance on organisms, such as the effect of invasive alien species on native communities [4] or of global warming on species distributions [5]. Because these factors may also coincide, it is important to consider the simultaneous impact of multiple factors on organisms’ phenotypes to uncover complex relationships linking phenotypes and the environment [6].

To assess the potential of organisms to cope with anthropogenic changes, it is necessary to examine not only the functional traits and variation in these traits but also their genetic underpinnings [7]. Adaptation may be mediated by changes in the genome sequence and in the gene expression level [8, 9]. An integrated approach linking phenotypic variation and gene expression would go one step further for detecting the genes and pathways related to the traits linked to fitness and involved in adaptation.

Numerous studies have documented the effects of multiple environmental factors on life history traits. Examples of multivariate analyses revealed how temporal and/or spatial environmental variations shaped life history traits in ectotherms [10–13]. This approach is particularly important for organisms with complex life cycles whose development through different developmental stages strongly depends on the environmental conditions [14]. Some studies have also incorporated human-related factors and provided evidence for their effects on phenotype, such as selection for different wing shapes along an urbanization gradient in mosquitoes [15] and pollutant-based selection for body size and mass in beetles [16]; similar studies were also conducted in crustaceans [17] and in plants [18].

Moreover, there is increasing interest in understanding how environmental changes drive genetic adaptation of the observed phenotypic changes [19]. The genetic basis of adaptation of organisms to anthropogenic changes has been particularly studied in the context of rapid urbanization [20, 21]. However, the transcriptomic response of organisms when exposed to anthropogenic factors has rarely been evaluated in response to herbicide [22] or in response to an increase in temperature matching the IPCC predictions [23–25]. In general, changes in gene expression caused by a small increase in temperature tended to be associated with major metabolic functions rather than with stress signalling pathways [23]. However, candidate genes underlying phenotypic differentiation in response to human-induced ecological stressors remain largely unknown. Here, we proposed to study differences in gene expression along both environmental and phenotypic variables and focused on their overlap to identify a set of candidate genes. Such information would provide valuable insights into the direction of physiological and developmental changes of an organism in response to a combination of different anthropogenic factors.

Here, we studied the individual and combined effects of temperature and invasive alien predators on both phenotypic and gene expression levels. In general, a rise in temperature, but below the thermal optimum, increases growth and development rates in ectotherms [26]. Previous studies indicated that the mere presence of a predator cue might affect developmental traits in potential prey [27]. Furthermore, the effect of warming temperature may influence the response of prey to a predator cue, e.g., by an additional reduction in growth [28] or mass at emergence [29], making these two variables particularly important in shaping predator–prey interactions in a warming world. However, little is known about how these factors affect the phenotype and expression of underlying genes. For this, we used a temperate damselfly, *Ischnura elegans*. Field-sampled individuals from replicated high- (southern Sweden) and central-latitude (southern Poland) ponds were used to consider the existing variability in life-history strategies linked to seasonal time constraints [30, 31]. Damselfly larvae were exposed to experimental treatments in a full factorial design: current (20 °C) and mild warming temperature (24 °C) corresponding to the average temperature increase by the end of the century (SSP8.5 scenario; IPCC 2021; [32]) crossed with the presence or absence of an invasive alien predator cue released by the spiny-cheek crayfish, *Faxonius limosus* [33]. The crayfish has co-occurred with Polish *I. elegans* populations for several decades but has not yet been reported in Scandinavia, including Sweden [34, 35]. Using phenotypic measures and transcriptomic analysis, we aimed to identify genes and metabolic pathways underlying the phenotypic response to a combination of warming and invasive alien predator cues. We specifically asked (1) how a set of life history traits correlates with a set of environmental factors using univariate and multivariate statistics and (2) what are the candidate genes and metabolic pathways underlying the changes among the most differentiated individuals regarding their phenotype and environment.

## Results

### Treatment effects on phenotypic traits

**Fig. 1:**
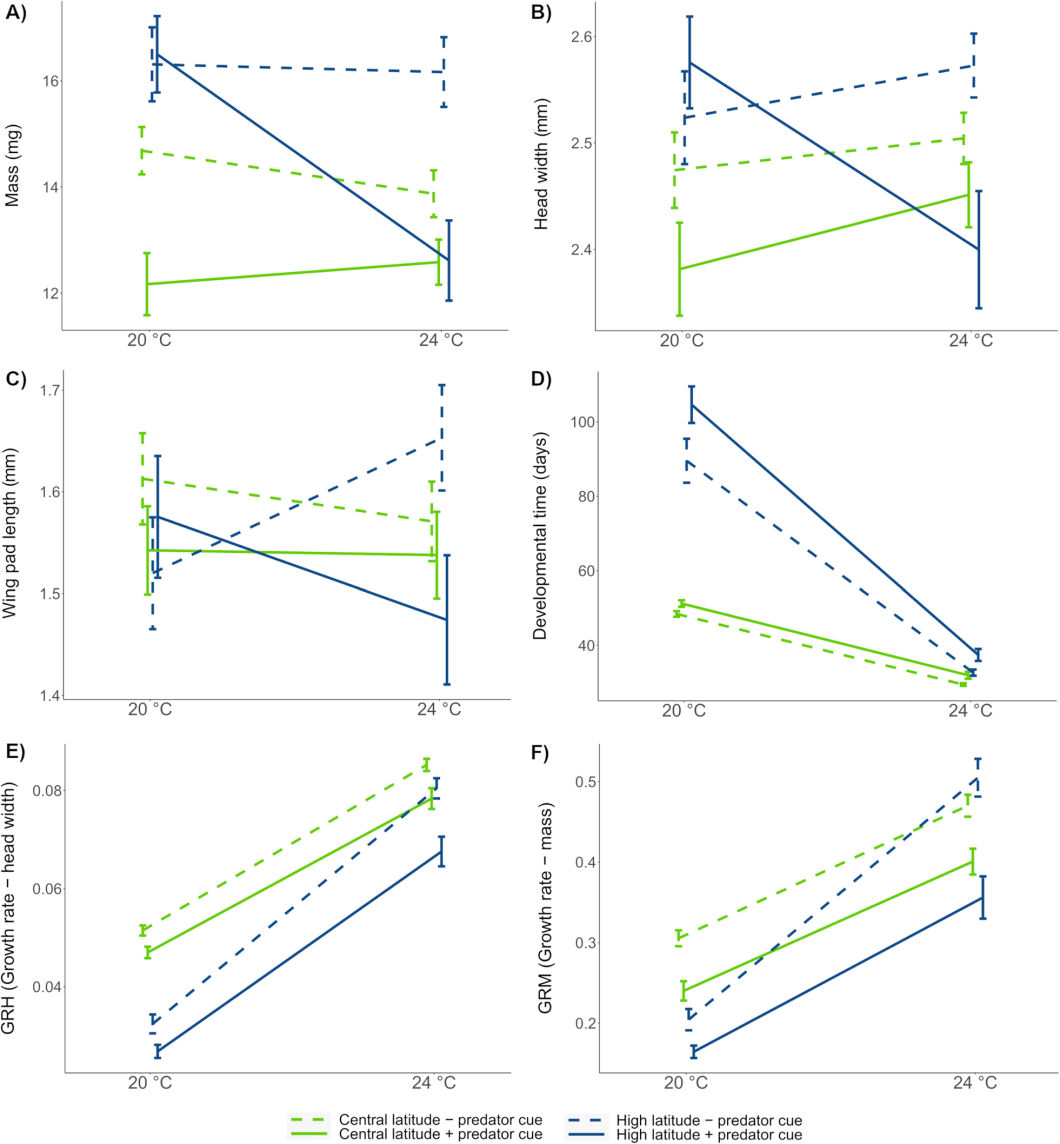
Effects of latitude (high vs. central), temperature (20 °C vs. 24 °C) and predator cue (presence vs. absence) on *I. elegans* larval **A** mass, **B** head width, **C** wing pad length, **D** developmental time, **E** growth rate based on head width, GRH, and **F** growth rate based on mass, GRM

First, we tested for the effects of environmental factors (latitude, temperature and predator cue) on each trait separately: wet mass (hereafter, mass), head width, wing pad length, growth rate based on mass (GRM) and growth rate based on body size (GRH) (Table 1). For mass, the three variables, temperature, latitude and predator cue, had significant effects with higher values at 20 °C, high latitude and no predator cue (Additional file 1: Table S1). The significant three-way interaction, latitude × temperature × predator cue, was mainly driven by the high-latitude individuals showing a decrease in mass at 24 °C in the presence of the predator cue (Fig. 1A). Similar trends were observed for head width, with the difference that temperature was not significant (Fig. 1B, Additional file 1: Table S1). We found no effect of the three variables on wing pad length, except a significant three-way interaction, latitude × temperature × predator cue, with the strongest difference found for the high-latitude individuals at 24 °C with longer wing pads in the absence of predator treatment (Fig. 1C). Central-latitude larvae developed within a shorter time than high-latitude larvae, but differences in development time were more pronounced in high-latitude individuals across different temperatures (interaction latitude × temperature; Table 1). Predator cues increased development time, especially at high latitudes (interaction latitude × predator) (Fig. 1D). The two measures of growth rate (GRH and GRM) showed similar patterns across treatments. The difference in GRH and GRM between the two latitudes was more pronounced at 20 °C than at 24 °C (interaction latitude × temperature; Fig. 1E and F). For GRM, high-latitude larvae grew the fastest, but only at 24 °C and in the absence of predator cues (interaction latitude × temperature × predator cue; Fig. 1F).

**Table 1:** Effect of latitude, temperature, predator cue and their interactions on phenotypic traits: mass, head width, wing pad length, development time (dev. time), growth rate based on head width (GRH) and mass (GRM)

Variables	Df	Mass	Head width	Wing pad length	Dev. time	GRH	GRM
		*p*	*p*	*p*	*p*	*p*	*p*
Latitude	1	< 0.001 (25.36)***	0.002 (9.76)**	0.759 (0.09)	< 0.001 (85.7)***	< 0.001 (56.1)***	< 0.001 (14.2)***
Temperature	1	0.009 (6.75)**	0.585 (0.30)	0.886 (0.02)	< 0.001 (1958)***	< 0.001 (1214)***	< 0.001 (356)***
Predator cue	1	< 0.001 (16.70)***	0.031 (4.66)*	0.138 (2.20)	< 0.001 (57.30)***	< 0.001 (45.95)***	< 0.001 (54.5)***
Latitude × temperature	1	0.038 (4.31)*	0.041 (4.19)*	0.533 (0.39)	< 0.001 (213.43)***	< 0.001 (57.49)***	< 0.001 (17.2)***
Latitude × predator cue	1	0.718 (0.13)	0.690 (0.16)	0.984 (0.00)	0.010 (6.72)**	0.071 (3.27	0.244 (1.36)
Temperature × predator cue	1	0.121 (2.40)	0.074 (3.19)	0.126 (2.35)	0.701 (0.15)	0.114 (2.49	0.019 (5.47)*
Latitude × temperature × predator cue	1	0.004 (8.40)**	0.019 (5.54)*	0.038 (4.32)*	0.657 (0.20)	0.348 (0.88	0.012 (6.35)*

The effects of random factors are not shown

Table shows *p* values and Wald Chi-squared statistic (in parentheses) for each variable. Significance is indicated in bold by ***p < 0.001, **p < 0.01, *p < 0.05

### Multivariate analysis

We further explored the relationship between our sets of phenotypic and environmental variables using canonical correlation analysis (CCA). CCA consists of finding the optimal combination of the seven phenotypic traits (canonical variate of the phenotype; CCp) and of the three environmental traits (canonical variate of the environment; CCe) so that the canonical correlation between the CCp and the CCe is maximized. Canonical correlation analysis (CCA) revealed three significant canonical correlations explaining 76% of the total variation (Table 2). After considering the variance explained by the first canonical correlation, the two remaining correlations were significant and explained 27% of the variance. After considering the variance explained by the first and second canonical correlations, the third correlation was also significant and explained 7% of the variance.

**Table 2:** Statistics and significance of the three canonical correlations together (1–3), of the second and third canonical correlation excluding the first one (2–3) and of the third canonical correlation only (3–3)

Canonical correlation	Wilks’ lambda	Rc	Rc^2^	F approx.	Df_num_	Df_den_	*p*
1–3	0.16	0.87	0.76	44.86	15	729	**< 0.001**
2–3	0.67	0.52	0.27	14.50	8	530	**< 0.001**
3–3	0.93	0.27	0.07	7.14	3	266	**< 0.001**

The table shows the Wilks’ lambda statistic, canonical correlation coefficient (Rc), proportion of shared variance (Rc^2^), F-approximation for Wilks’ lambda (F-approx.), numerator and denominator degrees of freedom (Df_num_ and Df_den_) and *p* value based on F-approximation (significant correlations are indicated in bold)

The loading and cross-loading coefficients reported in Table 3 indicate the contribution of each phenotypic and environmental variable to each canonical correlation. For the first canonical correlation, developmental time and GRM for the phenotypic variables and latitude and temperature for the environmental variables contributed the most to the correlation. At 24 °C and for the central latitude individuals, developmental time decreased and GRM increased. The second canonical correlation depicted a relationship between developmental time, mass and head width, and predator cue and latitude. In the presence of a predator cue and for central latitude individuals, mass and development time decreased, whereas head width increased. The last canonical correlation revealed that in the presence of a predator cue, both mass and GRM decreased.

**Table 3:** Canonical loadings and cross-loadings for the three canonical correlations

	1st canonical correlation		2nd canonical correlation		3rd canonical correlation	
	Loadings	Cross-loadings	Loadings	Cross-loadings	Loadings	Cross-loadings
*Canonical variable of phenotypic traits (CCp)*						
Mass	− 0.26	− 0.23	**− 0.58**	**− 0.30**	**− 0.42**	**− 0.12**
Head width	0.12	0.10	**0.39**	**0.20**	0.03	0.01
Wing pad length	− 0.00	− 0.00	0.09	0.05	0.28	0.08
Dev. time	**− 0.86**	**− 0.75**	**− 0.32**	**− 0.17**	0.23	0.06
GRM	**0.87**	**0.75**	− 0.22	− 0.12	**− 0.34**	**− 0.09**
*Canonical variable of environmental traits (CCe)*						
Latitude	**− 0.36**	**− 0.31**	**− 0.89**	**− 0.46**	0.28	0.08
Predator cue	− 0.19	− 0.16	**0.37**	**0.19**	**0.91**	**0.25**
Temperature	**0.93**	**0.81**	− 0.26	− 0.13	0.24	0.07

The phenotypic traits are mass, development time (dev. time), head width, wing pad length, and growth rate based on mass (GRM). Variables were considered significant (in bold) when canonical loadings were greater than |0.30|

### Gene expression analysis

To reveal genes and metabolic pathways underlying the associations between the phenotypic and environmental variables, we conducted two types of gene expression analyses for each significant canonical correlation previously identified-one with the most differentiated individuals of the canonical variable of phenotypic traits (CCp) and one with the most differentiated individuals of the canonical variate of the environment (CCe) (Additional file 2: Table S2). Candidate genes were those differentially expressed in the same direction in the two types of analyses.

### First canonical correlation

Gene expression analysis revealed a total of 1301 differentially expressed genes (DEGs) associated with the canonical variable of phenotypic traits (CCp1), with 781 DEGs upregulated and 520 downregulated in individuals with high GRM and short developmental time. We found 1592 DEGs associated with the canonical variate of the environment (CCe1), with 648 DEGs upregulated and 944 downregulated in individuals from central latitudes raised at 24 °C. Between the two sets of upregulated DEGs (781 and 648), 385 DEGs overlapped, and between the two sets of downregulated DEGs (520 and 944), 320 DEGs overlapped (Additional file 3: Table S3).

Gene ontology (GO) term enrichment analysis of the 385 DEGs upregulated in individuals from central latitudes raised at 24 °C with high GRM and short developmental time revealed only two enriched GO terms for cellular components related to membrane localization (Additional file 3: Table S3C). Because no enriched GO terms were found for biological process, we complemented the GO term enrichment analysis with a functional categorization to gain insight into the role of these DEGs. For the biological processes, genes were classified in various developmental processes (anatomical structure development, reproduction), primary metabolism and stress (Additional file 3: Table S3D).

**Fig. 2:**
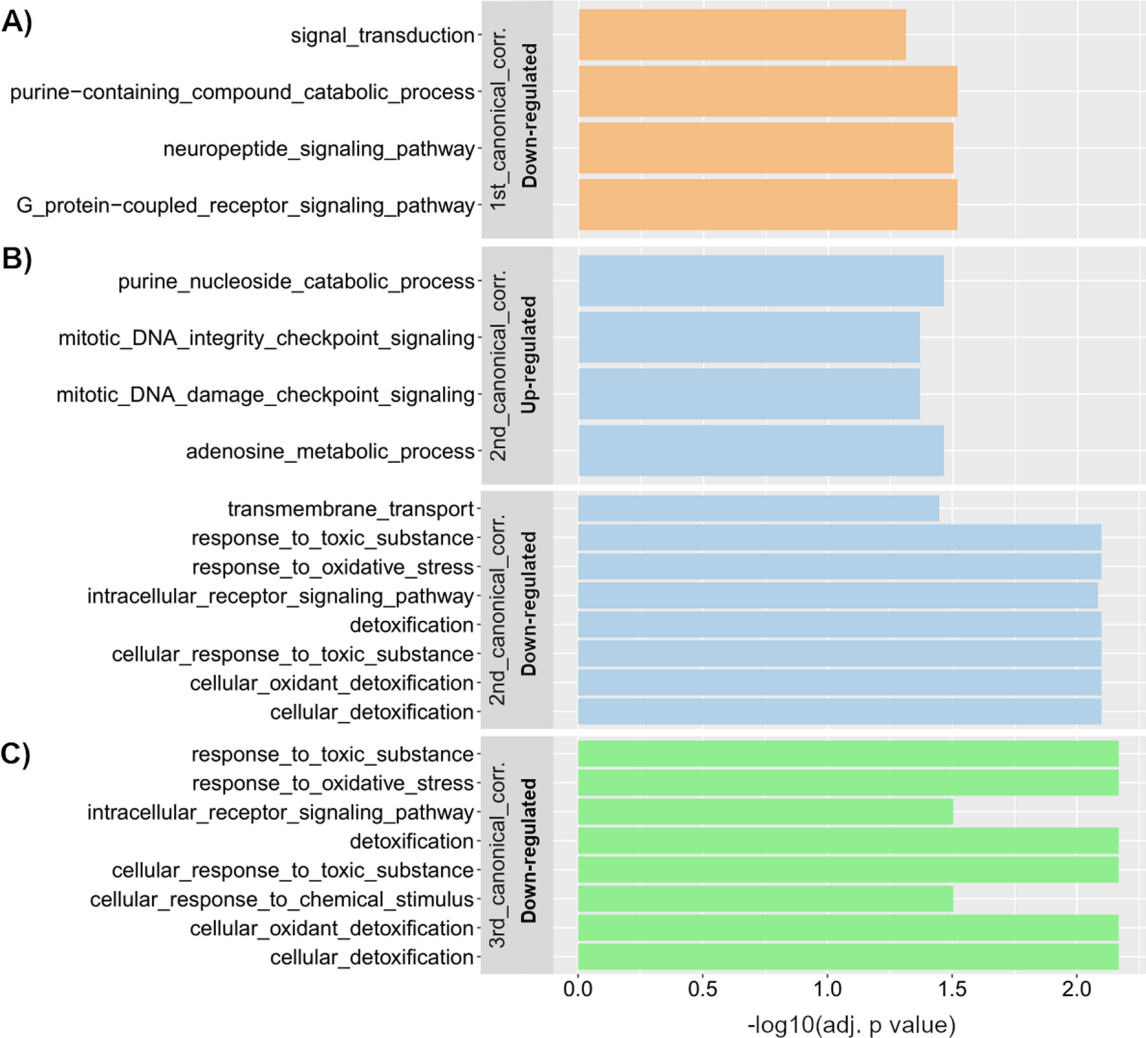
Enriched GO terms for biological process for **A** the 1st, **B** the 2nd and **C** the 3rd canonical correlation. More details on the GO term enrichment analysis for the 1st, 2nd and 3rd canonical correlations are provided in Additional files 3, 4, 5: Tables S3, S4 and S5, respectively. For the 2nd canonical correlation, 24 enriched GO terms in the set of upregulated genes were enriched due to only two genes. For clarity, we only show a small subset of the enriched GO terms. The full list of enriched GO terms is shown in Additional file 4: Table S4C

GO term enrichment analysis of the 705 downregulated DEGs revealed four enriched GO terms for biological processes related to signal transduction and one for cellular components related to the extracellular region (Fig. 2A, Additional file 3: Table S3C).

### Second canonical correlation

For CCp2, we found 124 DEGs that were upregulated and 681 that were downregulated in individuals with shorter developmental times, lower mass and high values for head width. For CCe2, we found 252 upregulated DEGs and 928 downregulated DEGs in central-latitude individuals exposed to the predator cue. We found that 62 DEGs overlapped between the two sets of upregulated DEGs and 634 DEGs overlapped between the two sets of downregulated DEGs (Fig. 2B; Additional file 4: Table S4).

GO term enrichment analysis of the 62 DEGs upregulated in both CCp2 and CCe2 revealed 24 enriched GO terms for biological processes associated with mitotic activity, purine and adenosine metabolism, and DNA repair. Two genes were highlighted of these 24 enriched GO terms: *ADENOSINE DEAMINASE 2-like* and *NIBRIN*.

GO term enrichment analysis of the 634 DEGs downregulated in both CCp2 and CCe2 revealed eight enriched GO terms for biological processes mostly related to oxidative stress. For the cellular component, enriched GO terms were associated with membranes. For molecular function, enriched GO terms were related to cuticle and chitin modification, transporter activity, oxidoreductase activity, serine hydrolase and to heme and tetrapyrrole binding.

### Third canonical correlation

For CCp3, we found 550 upregulated DEGs and 682 downregulated DEGs in individuals with lower GRM and lower mass. For CCe3, we found 309 upregulated DEGs and 357 downregulated DEGs in individuals exposed to the predator cue. Between the two sets of upregulated DEGs, 58 DEGs overlapped, and between the two sets of downregulated DEGs, 278 DEGs overlapped (Additional file 5: Table S5).

GO term enrichment analysis of the 58 DEGs upregulated in both CCp3 and CCe3 revealed three enriched GO terms for cellular components related to reproductive cells and seven enriched GO terms for molecular functions related to different activities, such as oxidoreductase or myosin binding. We complemented the GO term enrichment analysis with a functional categorization to identify biological processes in which the DEGs were involved. Functional categorization indicated a role in general metabolic and cellular processes and in reproduction.

GO term enrichment analysis of the 278 DEGs upregulated in both CCp3 and CCe3 revealed eight enriched GO terms for biological processes mostly related to oxidative stress (Fig. 2C); five enriched GO terms for cellular components associated with membranes and egg chorion; and nine enriched GO terms for molecular functions associated with cuticle modification, antioxidant activity and heme and tetrapyrrole binding.

## Discussion

Our results shed light on complex associations involving mild warming and invasive alien predator stress that differently affect the phenotype of high- and central-latitude individuals. Transcriptome analysis further revealed relevant metabolic pathways underlying these associations related to larval anatomy and development, reproduction or oxidative stress.

### Effects of environmental variables on phenotypic traits

The first canonical correlation revealed a shorter development time and faster growth rate in central-latitude individuals when exposed to mild-warming temperatures. These results matched the general pattern that ectotherms decrease development time and increase growth rate as temperature increases, but only until a certain temperature threshold [36], which was also demonstrated in damselflies, including *I. elegans* [31]. Previous studies also found a higher growth rate at low latitudes than at high latitudes [31, 37], supporting our results for this trait. Faster growth in central- than high-latitude populations might be explained by latitude-specific voltinism, with central-latitude damselflies completing, on average, more generations per year than high-latitude damselflies [30]. Such variable voltinism across latitudes increases seasonal time constraints in central-compared to high-latitude populations. Finally, a significant temperature-by-latitude interaction indicated that high-latitude populations had the steepest thermal reaction norm in development time and growth rate, yet central-latitude populations had the shortest development and the fastest growth. A strong thermal response in high-latitude damselflies might be caused by both latitudinal and voltinism compensation in development and growth, resulting in a relatively large final size and mass. Different life-history strategies related to latitude and voltinism affect larval development and growth in Odonata [38], and our current results confirm the complexity of latitude-, temperature- and voltinism-driven larval responses.

The second canonical correlation indicated that in the presence of a predator cue, for central-latitude individuals, mass and development time decreased, whereas head width (proxy of structural body size) increased. Previous studies on the effects of predators on *I. elegans* showed predator-specific effects on various traits. For example, exposure to the native dragonfly cue increased the growth rate independently of the latitude of origin [31]. Exposure to the invasive alien spiny-cheek crayfish cue reduced the egg development time compared with exposure to cues from other native and nonnative predator species [39]. Hence, the use of the spiny-cheek crayfish may lead to different responses in coping with the predator cue, which might depend on damselfly co-occurrence with the more or less phylogenetically related predator species [40]. Thus, shorter development time in central-latitude individuals may be part of an avoidance strategy to reduce the time of exposure prior to emergence to their nonnative aquatic predator, but at the cost of having a lower mass [39, 41]. Regarding larval size, some studies on other damselfly species found positive correlations between body size and swimming speed as a way to escape predators [42, 43]. However, the opposite pattern was found in *I. elegans* when larvae were exposed to a dragonfly predator [44], indicating that the effects of predator‒prey interactions are species dependent.

The last canonical correlation revealed that in the presence of a predator cue, both mass and GRM decreased, suggesting that a predator stress effect was shared by the different latitudes. For the effects of a predator cue on both mass and GRM, one explanation may be reduced foraging activity in the presence of a predator cue, as shown in other insect species [45]. However, no difference in food intake was reported in *I. elegans* when larvae were exposed to a predator [31]. Instead, the results pointed to differences in resource allocation. Another explanation may involve costs at the physiological level. There is evidence that predator stress alters the physiology and metabolism of insects [46] regarding fat content reduction [47], and may come at the cost of having a smaller size at emergence [48]. Despite the fact that the spiny-cheek crayfish has not yet been reported in high latitude sites, the mere presence of crayfish cues caused a short-term phenotypic response in *I. elegans* larvae. Indeed, our results suggest nonlethal effects of the invasive crayfish, especially when combined with increased temperature, for damselfly traits linked to fitness-mass and size reduction in high-latitude populations. This finding does not support recently published results based on meta-analysis where the authors stated that, on average, a couple of hundreds of generations are needed for prey to recognize nonnative predators [40]. However, previous prey experience with phylogenetically related predator species, here noble crayfish (*Astacus astacus*), might enable predator cue recognition. Our results may have implications for understanding the response of damselfly populations to the current spread of the spiny-cheek crayfish in Europe in general and in high-latitude habitats in particular.

### Gene expression analysis

Analysis of gene expression differences associated with the first canonical correlation revealed a relationship between latitude and increasing temperature on the rate of growth and development. Genes upregulated in individuals from central latitudes raised under mild warming and that had faster growth and shorter development were involved in metabolic (nitrogen compounds) and cellular (communication and transport) processes and in the development of anatomical structures. Although some genes involved in similar pathways were also downregulated in these individuals, our results pointed to modifications in primary metabolism and were consistent with differences in developmental rate between the two latitudes, as observed at the phenotypic level. These results are consistent with a previous study on *I. elegans*, where the authors found numerous genes related to metabolic pathways and to protein biosynthesis that were differentially expressed across latitudes and mild warming temperatures [23]. In general, rapid protein turnover and a high metabolic rate are strongly associated with an increase in temperature [49]. Across latitudes, variations in environmental variables such as temperature and the length of the growth season often lead to different life history strategies [50], and these strategies shape traits, such as growth and development [51], and behaviour, such as locomotor activity [52, 53], also confirming our observations at the phenotypic level.

For the second canonical correlation, only a few genes were upregulated in individuals from central latitudes exposed to the predator cue, with shorter developmental time, lower mass and larger head width. These genes were mostly related to general developmental processes. For example, two genes were responsible for several enriched GO terms for mitotic activity and DNA repair: *ADENOSINE DEAMINASE 2-like* is involved in the immune system, which is important for larval development and survival in *Drosophila* [54] and may have a similar role in *I. elegans,* and *NIBRIN* is involved in genome integrity [55]. In contrast, enriched GO terms for the downregulated genes were mostly related to oxidative stress, indicating differential stress responses between the two latitudes when under predator stress. The fact that the genes involved in oxidative stress tended to be downregulated in central latitudes may cause some phenotypic and developmental differences compared to their high-latitude counterparts. Indeed, exposure to a predator may have profound effects on insect physiology and the general stress response, particularly increased oxidative stress [46]. Oxidative stress is an important component of the signalling pathways in response to a predator with downstream effects on development and fitness, including growth, fecundity and survival. Empirical studies focusing on the activity of specific antioxidant enzymes showed reduced antioxidant activity when damselflies were exposed to a predator [56], which was also partly latitude-specific in our study. Because the invasive alien predator used in this study co-occurred with damselflies only at central latitudes, we may also expect differences in signalling pathways and/or sensory mechanisms between the studied latitudes. Based on our list of genes and GO terms, we were not able to clearly identify genes or pathways related to predator recognition. Notably, we found enriched GO terms for molecular function related to cuticula or chitin modifications. In insects, the cuticle has manifold roles, i.e., resistance to abiotic and biotic stress, sensory, body protection and support, and locomotion [57].

For the third canonical correlation, DEGs upregulated in individuals exposed to predator cues and having lower mass and GRM were involved in general metabolic pathways, including those linked to reproduction. In contrast, DEGs downregulated in these individuals were involved mostly in oxidative stress, confirming a previously shown reduced antioxidant activity under predation risk [56]. A reduced antioxidant activity may stem from energy cost or trade-off with the production of other stress proteins. A reduction in antioxidant activity may consequently induce an increase in reactive oxygen species that may impair physiological function and eventually cause a decrease in growth rate [58]. The predator cue effect on processes related to reproduction likely arose due to trade-offs between predator avoidance strategies and other vital functions, such as foraging [59, 60], as reported in other arthropods [61, 62]. These trade-offs are more likely to occur under chronic stress (the case in our study) than under acute stress [59, 61].

Finally, our study revealed transcriptomic differentiation between central- and high-latitude populations when exposed to a combination of mild warming and predator treatment. As we focused on the most differentiated individuals for the gene expression analysis, we might have not captured the full extent of the phenotypic variation at the gene expression level, finding only the most relevant genes or pathways (i.e., explaining an important part of the variance). In general, variations in gene expression may help cope with stressors and further provide material for long-term adaptation [9]. A previous study on *I. elegans* demonstrated genetic differentiation for growth rate along a latitudinal gradient that was mostly driven by differences in seasonal time constraint and voltinism [37]. Hence, some phenotypic and/or gene expression differences between central- and high-latitude populations may have arisen from genetic differentiation related to variable voltinism, i.e., cross-latitude constitutive differences in voltinism. However, as warming increases the length of the growth season and number of generations per year in the study species [29, 30] and might affect predator‒prey interactions [63], we anticipate further changes at the transcriptomic level in temperate populations of *I. elegans*.

## Conclusion

To conclude, our study addressed general questions on the effects of anthropogenic and natural factors in the context of climate change, biological invasion and latitude-specific seasonal time constraints in the damselfly *I. elegans*. We showed clear effects of these factors on phenotype and gene expression and their strong influence on traits linked to fitness. This may have wider implications on the biology of ectothermic organisms and may further impact their life history strategies in seasonal environments. Taken together, our results provide insights into how organisms may respond to future anthropogenic changes and are of particular interest from a conservation perspective.

## Methods

### Study species and collection

*Ischnura elegans* is a common damselfly in Europe, occurring from the southern half of Sweden to southern Italy and northern Spain [64]. High-latitude populations are uni-, semi or partivoltine, i.e., one, two or three years for completing one generation, respectively. However, a nonoverwintering generation, i.e., bivoltine cohort, has also been reported ([50]; Erik Svensson, pers. communication). Central-latitude populations are uni- and bivoltine [30, 50]. Previous studies indicated that the mere presence of a predator cue might affect *I. elegans* developmental traits [65, 66]. Adult females occur in three colour morphs: androchromatic, infuscan and infuscans-obsoleta [64, 67]. Copulating tandems were collected from two southern Sweden (hereafter, high latitude) and two southern Poland (hereafter, central latitude) ponds between 22 and 23 June 2021 (Additional file 1: Table S6) using a standard method [68]. The ponds were situated in rural areas, had similar sizes and depths, and contained populations of top freshwater predators. The two Swedish and Polish ponds were separated by 19.6 km and 67.9 km, respectively, and are not genetically isolated, as gene flow is generally high in *I. elegans* across its native range [37]. We sampled adult females from two different morphs, five androchromatic and five infuscan females, to avoid the interference of the mother’s morph type on offspring development [69]. Females were individually placed in plastic cups with perforated lids and wet filter paper for egg laying. Females were kept in a room at a temperature of 22 °C and natural daylight (photoperiod). In total, 40 clutches were used in the experiment, ten per location.

### Invasive alien predator species

The spiny-cheek crayfish, *F. limosus*, is a North American species introduced into Europe where it has been reported as an active colonizer [35]. *F. limosus* has locally co-occurred with the central latitude *I. elegans* for at least 50 years [70] and has not yet been recorded in Northern Europe, including Sweden [34]. Prior to the experiment, 20 *F. limosus* were collected from Kryspinów Lake in southern Poland (50° 3′ 0.461″ N, 19° 47′ 20.85″ E) and transported to the Institute of Nature Conservation, Kraków, Poland. Crayfish collection and housing were performed with permission from the Regional Directorate for Environmental Protection in Kraków (ref. OP.672.4.2021.GZ). For the treatment application, we kept *F*. *limosus* in aquariums (three crayfishes in 40 L of water) along with a control aquarium (40 L of water).

### Growth chamber experiment

Upon arrival at the laboratory, egg clutches (hereafter, maternal lines) were kept in a climate incubator (Pol-Eko ST 700) at 22 °C and a photoperiod of L:D 20:4 h. Once the eggs hatched, 10 individuals from each maternal line were randomly chosen for each of the four treatments. These groups of 10 individuals were reared in containers (size 17 × 12 cm, height 8 cm) filled with 700 mL of water and three nylon net strips, providing hiding space for larvae. Throughout the experiment, individuals were followed at the maternal line level. We did not report the sex of each individual larva. However, a clutch contained the same proportion of males and females in *I. elegans* [71], and we assumed that the final dataset was not sex biased. Our design involved two latitudes (high and central) × two temperatures (20 °C and 24 °C) × two predator treatments (presence and absence of a predator cue). We selected temperatures based on water surface temperature simulations in shallow parts of the collection ponds ([72]; estimation based on temperatures extracted from the years 1999–2009), on average temperatures do not exceed 20 °C (average temperature for the hottest month in Sweden = 19.4 °C; in Poland = 20.4 °C), and there were no significant differences in average temperature between high- and central-latitude ponds (Additional file 1: Fig. S1). Based on this and previous records of freshwater temperatures at sampled latitudes [73], we set two experimental temperatures, 20 °C and 24 °C. These temperatures represent current and predicted increased values by 2100, as modelled by the IPCC 2021 [32].

Containers were placed in two separate climate incubators corresponding to the two temperature treatments. Larvae were fed twice a day (week days) and once a day (weekend days) with laboratory-cultured *Artemia nauplii*. Every two days, a third of the water (233 mL) in every container was refilled with water either from the crayfish aquarium (containing predator cues) or from the control aquarium. Earlier studies have shown that the chemical cues of aquatic predators have an average half-life degradation time of ca. 36.5 h [74], which has been confirmed by previous experiments on nonconsumptive predator effects in damselfly larvae [65, 66, 75]. The experiment was complete when the first larvae per container moulted into a prefinal instar (hereafter, F-1). When two larvae emerged at the same time in the same container, we collected the two larvae to increase the sample size.

### Life history traits

For each container, we phenotyped the first larvae entering the F-1 stage (*N* = 2 latitudes × 2 ponds × 10 families × 2 temperatures × 2 treatments × 1–2 larvae = 272). The number of larvae per container that survived was noted at the end of the experiment. Due to logistic reasons, we reported the death of individuals raised at 20 °C only, but earlier studies showed no difference in larval survival across similar temperatures [73]. The number of larvae that had died in the 20 °C treatment did not significantly differ between high and central latitudes (Wald X^2^ = 2.06, Df = 1, *p* = 0.15). Every day before morning feeding, we checked for newly moulted F-1. Newly moulted individuals were placed in a separate incubator and were not fed (avoiding interference with weight or gene expression). At 14:00, each larva was photographed, and then head width, wing pad length and wet mass (hereafter, mass) were measured. After, F-1 were preserved in RNA later and kept at − 80 °C for the gene expression analysis. Larval development time was calculated as the number of days between hatching and entrance into F-1. The growth rate based on F-1 mass was calculated as mass/development time (GRM). The growth rate based on F-1 body size was measured as head width/development time (GRH). Head width is commonly measured for determining overall structural body size in odonates [76].

### Statistical analysis

First, we ran generalized linear mixed models for each of the six phenotypic traits to test for the effects of latitude, temperature, predator cue, and all of their interactions. Latitude, temperature, predator cue and their interaction were fixed factors; pond was treated as a random factor. The response variables, mass, head width, wing pad, GRM and GRH, were continuous variables (Gaussian distribution), and developmental time was count data (Poisson distribution). Head width and wing pad were log-transformed, and GRM and GRH were arcsin-transformed to approach a normal distribution. Each model was fitted using the function ‘glmmTMB’ in the ‘glmmTMB’ package [77] in R [78, 79], and *p* values were computed using the Wald chi-square test (Wald X^2^) implemented in the ‘car’ package [80].

Because natural selection does not act on the phenotypic traits separately, we took an approach that allows us to include the covariance between traits. We explored relationships between sets of phenotypic (mass, head width, wing pad, GRM and developmental time) and environmental variables (latitude, temperature and predator cue) using canonical correlation analysis (CCA), a multivariate statistical model that can accommodate both continuous and discrete variables. First, CCA creates new variables (‘canonical variates’) consisting of a linear combination of the seven phenotypic traits (canonical variate of the phenotype; CCp) and the three environmental traits together (canonical variate of the environment; CCe) so that the canonical correlation between the CCp and the CCe, measuring the strength of their relationship, is maximized [81]. Subsequent canonical correlations are extracted from the remaining variance. The maximum number of canonical correlations that can be extracted equals the number of variables in the smallest set (three in our case). Hence, the first canonical correlation extracted from the linear combination of the phenotypic and environmental traits, denoted CCp1 and CCe1, respectively, explained the greatest proportion of variance. The second canonical correlation consisting of a different combination of the phenotypic and environmental traits is denoted CCp2 and CCe2 and explained a lower proportion of variance, etc. for CCp3 and CCe3. To determine the relative importance of each original variable to the canonical correlation, we computed the canonical loadings and the canonical cross-loadings. Canonical loadings depict the linear correlation between variables and their respective canonical variables. Canonical cross-loadings represent the correlation of each observed phenotypic or environmental variable with the other canonical variate. For a large sample size (*N* > 200), canonical loadings are considered significant if they are greater than |0.30| [81]. CCA assumes independent variables within each set, and it is generally recommended to remove highly correlated variables to minimize multicollinearity issues [81]. We computed Spearman’s rank correlation coefficients between the six phenotypic variables (Additional file 1: Table S7); one variable, GRH, correlated strongly with the others (r > 0.9, *p* < 0.001) and was discarded from the analysis. The remaining phenotypic variables were transformed as for the generalized linear mixed model analysis to approach a normal distribution. Canonical correlation analysis was performed at the individual level (*N* = 272) using the package CCA in R [82]. We used the F-approximation of Wilks’ lambda to test for the significance of the canonical correlation coefficients using the ‘p.asym’ function (‘CCP’ package in R; [83]).

### Gene expression

For sequencing, we used the ten most differentiated individuals with respect to their phenotype and environment for each canonical correlation. Total RNA was extracted using RNAzol (MRC), and its integrity was assessed by agarose electrophoresis and on the Agilent 2100 Bioanalyzer. Libraries were prepared from up to 1 μg of the total RNA using NEBNext Ultra II Directional RNA Library Prep Kit Illumina (indexed with NEBNext Multiplex Oligos for Illumina (Dual Index Set 1 and Set2). Fragmentation time followed the protocol, resulting in insert sizes of ca. Then, 200 bp and 9 PCR cycles were used to amplify the libraries. The libraries were sequenced on DNBSEQ T7 (2 × 100 bp reads) at BGI. Sequencing generated between 20.3 and 40.4 million raw reads per individual. The data are available from the Sequence Read Archive under accession PRJNA899331. Reads were mapped using hisat2 2.1.0 [84] on the *I. elegans* reference genome using the corresponding annotation generated by the Darwin Tree of Life Project (https://www.darwintreeoflife.org/; project ID: PRJEB46264) [85]. We excluded the sex chromosome to avoid any biases because we did not distinguish between males and females. The number of reads mapped on each gene was counted with featurecounts 2.0.3 [86], and only the uniquely mapped reads were retained (between 12.9 and 29.3 million reads per individual). Differential gene expression analysis was performed using EdgeR v3.15 [87].

Gene expression analysis was performed on the output of the canonical correlation analysis. For each significant canonical correlation, we conducted two types of gene expression analyses, one with the canonical variate of phenotypic traits (CCp) and one with the canonical variate of the environment traits (CCe). For each canonical correlation, we extracted the canonical scores of each individual, depicting the contribution of each individual to the correlation, and individuals were sorted according to their canonical scores (Additional file 2: Table S2). For the first canonical correlation, we performed the first gene expression analysis between the ten individuals with the highest and the ten individuals with the lowest scores for CCp1 and a second gene expression analysis with the ten individuals with the highest and the ten individuals with the lowest scores for CCe1. Hence, we obtained two lists of differentially expressed genes, one underlying phenotypic difference and one underlying environmental difference. Then, genes were considered candidates underlying phenotypic trait differentiation along environmental variables if their gene expression overlapped across the two lists in the same direction. We repeated the same procedure with the second and third canonical correlations. Gene expression analysis was performed on the most differentiated individuals to ensure a sufficient number of differentially expressed genes were detected for the subsequent GO term enrichment analysis. *p* values were adjusted for multiple testing with the Benjamini and Hochberg false discovery rate correction (FDR), and the significance level was set at FDR < 0.05.

### Gene ontology

We created a custom Gene Ontology (GO) annotation. For each locus in the *I. elegans* genome, we retrieved the function and description from NCBI (https://www.ncbi.nlm.nih.gov/search/all/?term=ischnura%20elegans). Then, we extracted all genes described in insects with their respective GO terms from the UniProt database [88] and compared this gene list with that of *I. elegans* genes. If a gene in *I. elegans* had a similar function and name and was associated with the exact same GO terms in at least three distinct insect species, we assumed the *I. elegans* gene to be involved in the same metabolic pathways. With this method, among the 21,087 genes described in the *I. elegans* genome annotation, we were able to assign GO terms to 4807 of them. We performed a GO term enrichment analysis with BiNGO v3.0.3 software [89] in Cytoscape v3.5.1 [90] with the most recent gene ontology annotation downloaded from The Gene Ontology Resource [91]. GO terms were considered significantly enriched if the FDR-adjusted *p* value was < 0.05. For a few analyses, the GO term enrichment analysis did not return enriched GO terms, and we also performed a functional categorization where genes were grouped according to their GO annotations using WEGO [92].

## Supplementary Information

**Additional file 1. Fig S1:** Average surface temperature for each pond. **Table S1:** Least square mean values for each phenotypic and developmental trait. **Table S6:** GPS coordinates of each pond. **Table S7:** Spearman rank correlation coefficients between the phenotypic and developmental traits.  

**Additional file 2. Table S2:** Dataset used in this study.

**Additional file 3. Table S3:** Lists of differentially expressed genes and GO term enrichment analysis associated with the first canonical correlation.

**Additional file 4. Table S4:** Lists of differentially expressed genes and GO term enrichment analysis associated with the second canonical correlation.

**Additional file 5. Table S5:** Lists of differentially expressed genes and GO term enrichment analysis associated with the third canonical correlation.

## Data Availability

The sequence data that support the findings of this study are available from the Sequence Read Archive (https://www.ncbi.nlm.nih.gov/bioproject/899331) under accession PRJNA899331.
