# Supplementary material for: The effect of temperature and invasive alien predator on genetic and phenotypic variation in the damselfly Ischnura elegans: cross-latitude comparison
Source: Front Zool. 2023 Apr 10;20:13. doi: 10.1186/s12983-023-00494-z (PMC10084621; doi:10.1186/s12983-023-00494-z)
Supplement: Supplementary file 1 — Additional file 1. Fig S1: Average surface temperature for each pond. Table S1: Least square mean values for each phenotypic and developmental trait. Table S6: GPS coordinates of each pond. Table S7: Spearman rank correlation coefficients between the phenotypic and developmental traits. [file 12983_2023_494_MOESM1_ESM.docx]

**The effect of temperature and invasive alien predator on genetic and phenotypic variation in the damselfly *Ischnura elegans*: cross-latitude comparison**

Guillaume Wos, Gemma Palomar, Marzena Marszałek, Wiesław Babik, Szymon Sniegula

**Supplementary information**

**Fig. S1**. Average surface temperature estimated using Flake (Lake Model Flake 2009; estimation based on temperatures extracted from the years 1999-2009) for the two Swedish, Torups (SW) and Vallkärra (SW), and the two Polish, Zagorze (PL) and Niepolomice (PL), ponds. In addition, we also placed two dataloggers (at a depth of 40 cm): one in another pond in the locality of Niepolomice at 11 km from the ponds where damselflies were collected (on the edge of the same forest; year 2021-2022; GPS coordinates 50.012685 Lat.; 20.287012 Long. [Niepolomice (logger)]) and one in the pond in the locality of Torups (temperature recorded from 07.07.2022 to 19.10.2022 [Torups (logger)]).

The table below shows statistics of anova for A) the effects of latitude (high vs central latitude) on average temperatures estimated using Flake and B) for the effects of the method used to estimate temperature in the locality of Niepolomice between Flake (dark green curve) and the datalogger (dark red curve). No statistical difference was found neither between latitudes nor between the surface temperature estimated using Flake and the temperature measured with a datalogger at a depth of 40 cm.


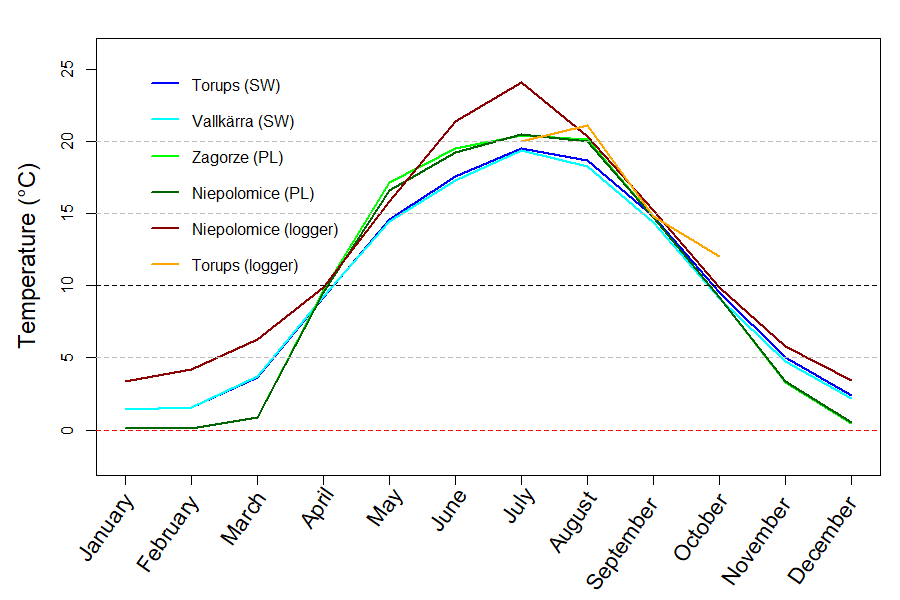


| **A)** |  |  | **B)** |  |  |
| --- | --- | --- | --- | --- | --- |
|  | **df** | **Temperature** |  | **df** | **Temperature** |
|  |  | *p* |  |  | *p* |
| **Latitude** | 1 | 0.949 (0.004) | **Flake vs. logger** | 1 | 0.528 (0.411) |

Table shows *p* value and *F* value in parentheses.

**Table S1**. Least square mean values and standard errors for each phenotypic and developmental trait and for each environmental variable.

|  | **Latitude** | | **Temperature** | | **Predator cue** | |
| --- | --- | --- | --- | --- | --- | --- |
|  | **Low-latitude** | **High-latitude** | **20 °C** | **24 °C** | **Absence** | **Presence** |
| Mass (mg) | 13.3 ± 0.327 | 15.5 ± 0.318 | 15.0 ± 0.33 | 13.9 ± 0.34 | 15.3 ± 0.33 | 13.6 ± 0.33 |
| Head width (mm) | 2.45 ± 0.02 | 2.52 ± 0.02 | 2.49 ± 0.02 | 2.49 ± 0.02 | 2.52 ± 0.02 | 2.46 ± 0.02 |
| Wing size (mm) | 1.57 ± 0.03 | 1.56 ± 0.03 | 1.56 ± 0.03 | 1.56 ± 0.03 | 1.59 ± 0.03 | 1.54 ± 0.03 |
| Developmental time (N days) | 40.5 ± 2.45 | 67.5 ± 2.52 | 74.5 ± 2.07 | 32.8 ± 2.14 | 50.5 ± 2.70 | 58.4 ± 2.73 |
| GRH (mm/day) | 0.07 ± 0.00 | 0.05 ± 0.00 | 0.04 ± 0.00 | 0.08 ± 0.00 | 0.06 ± 0.00 | 0.05 ± 0.00 |
| GRM (mg/day) | 0.353 ± 0.01 | 0.303 ± 0.01 | 0.23 ± 0.01 | 0.44 ± 0.01 | 0.37 ± 0.01 | 0.28 ± 0.01 |

**Table S6**. GPS coordinates of the different sampling localities

|  |  |  | **GPS coordinates** | |
| --- | --- | --- | --- | --- |
| **Country** | **Locality** | **Latitude** | **Lat.** | **Long.** |
| Poland | Niepolomice | Central | 50.10875 | 20.348707 |
| Poland | Zagórze | Central | 50.083352 | 19.39736 |
| Sweden | Torups | High | 55.564293 | 13.205614 |
| Sweden | Vallkärra | High | 55.738166 | 13.153274 |

**Table S7**. Spearman rank correlation coefficients between the phenotypic and developmental traits.

|  | Weight | Dev time | Head width | Wing size | GRW |
| --- | --- | --- | --- | --- | --- |
| Weight | 1 |  |  |  |  |
| Dev time | **0.24***** | 1 |  |  |  |
| Head width | **0.82***** | 0.07 | 1 |  |  |
| Wing size | **0.77***** | 0.02 | **0.84***** | 1 |  |
| GRW | **0.21***** | **-0.85***** | **0.29***** | **0.36***** | 1 |
| GRH | -0.11 | **-0.97***** | 0.09 | **0.13*** | **0.92***** |

Significance is indicated by *** p < 0.001, * p < 0.05.
